# Variable climatic conditions dominate recent phytoplankton dynamics in Chesapeake Bay

**DOI:** 10.1038/srep23773

**Published:** 2016-03-30

**Authors:** Lawrence W. Harding, Jr., Michael E. Mallonee, Elgin S. Perry, W. David Miller, Jason E. Adolf, Charles L. Gallegos, Hans W. Paerl

**Affiliations:** 1 Department of Atmospheric and Oceanic Sciences, University of California, Los Angeles, Los Angeles, California 90095, United States; 2 Interstate Commission on the Potomac River Basin, United States Environmental Protection Agency Chesapeake Bay Program Office, 410 Severn Avenue, Annapolis, Maryland 21403, United States; 3 Statistics Consultant, 2000 Kings Landing Road, Huntingtown, Maryland 20639, United States; 4 US Naval Research Laboratory, 4555 Overlook Ave., SW, Washington, D.C. 20375, United States; 5 Marine Science Program, University of Hawaii at Hilo, 200 W. Kawili Street, Hilo, Hawaii 96720, United States; 6 Smithsonian Environmental Research Center, 647 Contees Wharf Road, Edgewater, Maryland 21037, United States; 7 Institute of Marine Sciences, University of North Carolina at Chapel Hill, 3431 Arendell Street, Morehead City, North Carolina 28557, United States

## Abstract

Variable climatic conditions strongly influence phytoplankton dynamics in estuaries globally. Our study area is Chesapeake Bay, a highly productive ecosystem providing natural resources, transportation, and recreation for nearly 16 million people inhabiting a 165,000-km^2^ watershed. Since World War II, nutrient over-enrichment has led to multiple ecosystem impairments caused by increased phytoplankton biomass as chlorophyll*-a* (*chl-a*). Doubled nitrogen (N) loadings from 1945–1980 led to increased *chl-a*, reduced water clarity, and low dissolved oxygen (DO), while decreased N loadings from 1981–2012 suggest modest improvement. The recent 30+ years are characterized by high inter-annual variability of *chl-a*, coinciding with irregular dry and wet periods, complicating the detection of long-term trends. Here, we synthesize time-series data for historical and recent N loadings (TN, NO_2_ + NO_3_), *chl-a*, floral composition, and net primary productivity (NPP) to distinguish secular changes caused by nutrient over-enrichment from spatio-temporal variability imposed by climatic conditions. Wet years showed higher *chl-a*, higher diatom abundance, and increased NPP, while dry years showed lower *chl-a*, lower diatom abundance, and decreased NPP. Our findings support a conceptual model wherein variable climatic conditions dominate recent phytoplankton dynamics against a backdrop of nutrient over-enrichment, emphasizing the need to separate these effects to gauge progress toward improving water quality in estuaries.

The coastline of the United States is indented by a number of highly productive estuaries, connecting varied landscapes to receiving waters of the continental shelf. These land-margin ecosystems are characterized by significant seasonal to inter-annual variability of key properties and processes, reflecting a combination of anthropogenic and climatic influences^1^,^2^. Human activities, especially deforestation, expansion of agriculture and industry, urbanization, overfishing, and wastewater disposal have contributed to nutrient over-enrichment of estuaries^3^. Over the last half-century, steep increases in nutrient loadings led to increased phytoplankton biomass as chlorophyll*-a* (*chl-a*) in most but not all estuaries^4^,^5^, culminating in ecosystem impairments including low dissolved oxygen (DO), reduced water clarity, and harmful algal blooms (HAB), and prompting management actions to reverse cultural eutrophication^6^,^7^,^8^,^9^.

Chesapeake Bay on the mid-Atlantic coast is a prominent example of an estuary exhibiting symptoms of nutrient over-enrichment (Fig. 1). The bay is a large, eutrophic ecosystem where post-World War II increases of nitrogen (N) and phosphorous (P) loadings^10^ stimulated increases of phytoplankton biomass^2^,^11^,^12^ and net primary productivity (NPP)^13^, supporting high rates of microbial metabolism and subsequent bottom-water anoxia during summer^14^,^15^,^16^. The impact of nutrient over-enrichment is documented as a steep, upward trajectory of *chl-a* after the early 1950s, with 5- to 10-fold increases in seaward regions of the bay, and 1.5- to 2-fold increases landward^2^,^11^,^12^. Superimposed on the long-term trend of *chl-a* that signals cultural eutrophication, detailed analyses suggest a shift has occurred wherein variable climatic conditions dominate recent phytoplankton dynamics^17^,^18^,^19^,^20^,^21^.

Past studies linked phytoplankton dynamics to climatic conditions for a variety of estuaries, including Chesapeake Bay^22^,^23^; the Hudson River^24^,^25^; northern San Francisco Bay^26^; the Neuse River estuary^27^,^28^; the Loire River estuary^29^; and the lower Chesapeake Bay and adjacent continental shelf^30^. Nearly 30 years ago, Malone *et al.*^22^ reported the magnitude of freshwater flow from the Susquehanna River explained inter-annual variability of phytoplankton biomass in Chesapeake Bay. Aircraft remote sensing of ocean color later confirmed this view, relating the timing, position, and magnitude of the spring diatom bloom to climatic conditions^18^. Shipboard measurements of NPP showed that climatic effects on hydrology accounted for inter-annual variability of annual integral production (AIP)^13^. Predominant sea-level pressure (SLP) patterns supported a synoptic climatology that related freshwater flow to climatic conditions^31^, explaining inter-annual variability of *chl-a*, NPP, and AIP^18^,^32^,^33^.

Here, we synthesize long-term data on phytoplankton dynamics in Chesapeake Bay to distinguish trends reflecting cultural eutrophication from variability imposed by climatic conditions. Historical and recent data show mean annual surface *chl-a* peaked in the mid-1980s, followed by years with high spatio-temporal variability associated with by variable climatic conditions. The past several decades feature irregular dry and wet periods that account for inter-annual variability of freshwater flow and nutrient loadings to the bay, generating commensurate variability of water-quality properties^2^. We bring together extensive data for the period 1950–2015, using complementary measures including *chl-a*, floral composition, and NPP to show that variable climatic conditions dominate recent phytoplankton dynamics in the bay. Our approach develops and applies statistical models to long-term data assembled from hydrological sampling, shipboard monitoring, and aircraft remote sensing. These findings have implications for understanding phytoplankton dynamics in estuarine ecosystems subject to contemporaneous forcing by nutrient over-enrichment and climatic conditions.

## Results

### Freshwater flow, nitrogen loading

Annual freshwater flow from the Susquehanna River (SRF), the largest river entering Chesapeake Bay, was highly variable for calendar years from 1945–2015, reflecting irregularly spaced dry and wet years (Fig. 2). Successive decades of low and high annual SRF prevailed in the 1960s and 1970s, corresponding to protracted drought and flood conditions, respectively. Since the 1970s, flow extremes have been frequent, exemplified by several high-flow years after the mid-1990s, e.g., 1996, 2003, 2004, and 2011, all well above the 75^th^ percentile of annual SRF from 1945–2015 of 40.7 (×10^9^) m^3^ yr^−1^ (Fig. 2). Despite high inter-annual variability of annual SRF, there was no significant monotonic trend from 1945–2015 based on a Mann Kendall test (p > 0.778). Observed TN and NO_2_ + NO_3_ loadings (Fig. 3, filled dots, SE bars) increased significantly from 1945–2012, approximately doubling by the early 1980s, and characterized by high inter-annual variability over the last 30+ years.

Inter-annual variability of TN and NO_2_ + NO_3_ loadings (see Methods for data sources) resembled that of annual SRF as concentrations measured at the gaging station varied much less than freshwater flow^2^. We accounted for this effect of climatic conditions by developing generalized additive models (GAM, see Methods) to obtain flow-adjusted TN and NO_2_ + NO_3_ loadings (Fig. 3, solid lines). Additional details of the statistical approach to generate flow-adjusted outputs for loadings and water-quality properties are given by Harding *et al.*^2^. Annotations on the historical trajectories of TN and NO_2_ + NO_3_ loadings depicted in Fig. 3(a) correspond to the post- World War II period (1) wherein flow-adjusted TN loadings from GAM increased from 28 to 64 (×10^6^) kg yr^−1^ from 1945–1980 (+121%), leveling off around 1980 (2), and decreased loadings (3) from 66 to 53 (×10^6^) kg yr^−1^ from 1981–2012 (−19.2%). NO_2_ + NO_3_ loadings increased from 21 to 41 (×10^6^) kg yr^−1^ from 1945–1980 (+89.8%), and decreased from 41 to 39 (×10^6^) kg yr^−1^ from 1981–2012 (−5.3%). These flow-adjusted TN and NO_2_ + NO_3_ loadings confirmed steep increases that culminated in nutrient over-enrichment, followed by gradual decreases after the implementation of nutrient-management strategies in the early 1980s.

### Phytoplankton biomass as chlorophyll-a (*chl-a*)

Phytoplankton biomass as mean annual surface *chl-a* increased in oligohaline (OH), mesohaline (MH), and polyhaline (PH) salinity zones from 1950–2015, reflecting long-term nutrient over-enrichment of the bay punctuated by successive drought – flood periods in the 1960s and 1970s (Fig. 4, solid dots, SE bars). Mean annual surface *chl-a* in the OH salinity zone was ~3.5 mg m^−3^ in the early 1950s, increased steeply to 4.5 to 9.3 mg m^−3^ in the 1960s, and reached maximum concentrations of 18 to 23 mg m^−3^ by the early 1970s. After the mid-1970s, surface *chl-a* in the OH salinity zone decreased by half, declining from >20 mg m^−3^ to relatively constant, recent concentrations that range from 9.1 to 11 mg m^−3^ from 2006–2015 (Fig. 4a).

Mean annual surface *chl-a* in the MH salinity zone increased from 0.78 to 0.89 mg m^−3^ in 1950–51 to 1.9 to 6.0 mg m^−3^ in the mid-1960s, reaching 3.5 to 10 mg m^−3^ by the mid- to late-1970s (Fig. 4b). The upward trajectory of surface *chl-a* in the MH salinity zone resembled that for the OH salinity zone early in the time series, except *chl-a* has continued to increase gradually in the MH salinity zone after the 1980s, showing inter-annual variability from 8.3 to 13 mg m^−3^.

Consistent with the increases of mean annual surface *chl-a* in OH and MH salinity zones, concentrations in the PH salinity zone increased from 0.39 to 0.83 mg m^−3^ in 1950–51 to 1.4 to 2.0 mg m^−3^ by the late-1960s, and ranged from 4.1 to 8.1 mg m^−3^ in the late-1970s (Fig. 4c). After the 1980s, surface *chl-a* in the PH salinity zone has shown high inter-annual variability, ranging broadly from 4.0 to 11 mg m^−3^ through the mid-2000s. Recent values of mean annual surface *chl-a* have remained fairly constant, ranging from 5.4 to 7.2 mg m^−3^ from 2006–2015.

Time-series data for mean annual surface *chl-a* showed inter-annual variability resembling that of annual SRF and unadjusted TN and NO_2_ + NO_3_ loadings (compare Figs. 2, 3, 4), prompting us to account for climatic conditions using GAM to derive flow-adjusted surface *chl-a*. GAM for surface *chl-a* in OH and MH salinity zones (Fig. 4a,b, solid lines) explained >70% of the variances with R^2^ = 0.702 and 0.814, respectively (Table 1). GAM for surface *chl-a* in the PH salinity zone explained >85% of the variance with R^2^ = 0.864 (Fig. 4c; Table 1). Comparison of observed, fitted, and flow-adjusted values of mean annual surface *chl-a* in OH, MH, and PH salinity zones confirmed a strong effect of climatic conditions on phytoplankton biomass.

Long-term trends from 1950–2015 computed from flow-adjusted *chl-a* (Fig. 4) showed >100% increase in the OH salinity zone, and >1000% increases in MH and PH salinity zones. Decadal trends revealed the increase of flow-adjusted *chl-a* in the OH salinity zone reversed in the 1970s, while upward trends continued in MH and PH salinity zones after 1980, although the trajectories were shallower than from 1950–1980. Harding *et al.*^2^ provided additional details on long-term trends of water-quality properties based on flow-adjusted data.

Spatio-temporal distributions of surface *chl-a* based on data from *in-situ* monitoring and aircraft remote sensing of ocean color were distinct for dry and wet years (Fig. 5a,b). Surface *chl-a* from *in-situ* monitoring was higher in MH and PH salinity zones during wet years than the LTA or dry years, contrasted with the OH salinity zone where wet years had lower surface *chl-a* (Fig. 5a). Remotely sensed *chl-a* in spring showed a similar response as *in-situ chl-a* to climatic conditions, with higher remotely sensed *chl-a* during wet years than the LTA or dry years (Fig. 5b)^18^. Restricted aircraft operations limited retrievals for the OH salinity zone, accounting for limited matches with *in-situ chl-a* for the uppermost bay (Fig. 5a,b).

### Floral composition

Climatic conditions affected floral composition as the proportions of *chl-a* in major taxonomic groups (*f_chl-a*^*taxa*^). Diatoms were the predominant taxa in the bay, with significant contributions from dinoflagellates, cryptophytes, and cyanobacteria throughout the study period. The proportion of diatoms in the flora was affected by climatic conditions in all seasons with higher *f_chl-a*^*diatom*^ during wet years than the LTA or dry years (Fig. 6). Diatoms were the most abundant taxa in spring with 4% higher *f_chl-a*^*diatom*^ during wet years than the LTA and 12% higher than dry years. Cryptophytes were less abundant in spring during wet years with 3 to 5% lower *f_chl-a*^*crypto*^ than the LTA or dry years, while dinoflagellate abundance was unchanged by climatic conditions, with nearly identical *f_chl-a*^*dino*^ during wet, LTA, and dry years (Fig. 6a–c). Diatoms and dinoflagellates were more abundant in summer during wet years than the LTA or dry years. *f_chl-a*^*diatom*^ was 4% higher during wet years than the LTA and 7% higher than dry years, while *f_chl-a*^*dino*^ was 6% higher during wet years than the LTA and 7% higher than dry years. Cyanobacteria were less abundant in summer during wet years with 6% lower *f_chl-a*^*cyano*^ than the LTA and 10% lower than dry years (Fig. 6d–f). Diatoms and cryptophytes were the most abundant taxa in fall and reflected climatic conditions as in other seasons. *f_chl-a*^*diatom*^ in fall during wet years was 6% higher than the LTA and 12% higher than dry years, while cryptophytes were less abundant in fall during wet years with 5% lower *f_chl-a*^*crypto*^ than the LTA and 15% lower than dry years (Fig. 6g–i). ANOVA showed significant contributions by season, salinity zone, and year to the variances of *f_chl-a*^*taxa*^ for several major taxonomic groups, with highly significant (p ≪ 0.001) effects of climatic conditions on all but one group (Table 2).

Variable climatic conditions also affected cell size and floral composition, evident for consecutive dry (1995) and wet (1996) years as the cumulative proportion of cell volume (CV) vs. equivalent spherical diameter (ESD) and corresponding *f_chl-a*^*taxa*^ (Fig. 7). The cell-size distribution in spring during the wet year (1996) showed a shift to larger cells compared to the dry year (1995) (Fig. 7a,b). Differences of cell-size distributions were manifested as higher 10^th^ percentile, median, and 90^th^ percentile of ESD, and higher *f_chl-a*^*diatom*^ during the wet year (1996) than during the dry year (1995). Similar effects of climatic conditions were observed in summer, with a shift of the cell-size distribution to larger cells, shown as higher 10^th^ percentile, median, and 90^th^ percentile of ESD and higher *f_chl-a*^*diatom*^ during the wet year (1996) than during the dry year (1995) (Fig. 7c,d). A shift of cell-size distribution to larger cells during 1996 reflects the increased abundance of large, centric diatoms in MH and PH salinity zones where seasonal N-limitation is partly alleviated by higher SRF and increased N-loadings.

### Net primary productivity (NPP)

Annual cycles of euphotic-layer *chl-a* and NPP based on data from 1982–2004 revealed displaced maxima of biomass in spring and NPP in summer (Fig. 8). Mean euphotic-layer *chl-a* ranged from 40 to 100 mg m^−2^, with a spring maximum ~100 mg m^−2^ during wet years that was ~20% higher than the LTA or dry years. Consistent with the climatic effect on euphotic-layer *chl-a* in spring, we observed a summer mean >60 mg m^−2^ during wet years that was higher than the LTA or dry years. NPP showed a broad summer maximum during wet years, with a mean ~2250 mg C m^−2^ d^−1^, higher than the LTA of 2000 mg C m^−2^ d^−1^ and 1500 mg C m^−2^ d^−1^ during dry years.

Seasonal and salinity-zone means for salinity, *chl-a*, euphotic-layer *chl-a*, and NPP from 1982–2004 aggregated by climatic conditions showed strong contrasts for dry, LTA, and wet years (Table 3). Significant contributions to the variances of salinity, *chl-a*, euphotic-layer *chl-a*, and NPP by salinity zone, season, and climatic condition were documented by ANOVA (p < 0.01). *Chl-a* and euphotic-layer *chl-a* were higher in MH and PH salinity zones during wet years than the LTA or dry years, and NPP was also higher in these salinity zones during wet years. Opposite responses to climatic conditions occurred in the OH salinity zone than in MH and PH salinity zones. Plots of euphotic-layer *chl-a* and NPP versus salinity for OH, MH, and PH salinity zones illustrate these effects (Fig. 9). The lower salinities corresponding to wet years were accompanied by lower euphotic-layer *chl-a* and NPP in the OH salinity zone, while both measures of phytoplankton dynamics were higher in MH and PH salinity zones (see arrows, Fig. 9). This pattern reflected a combination of light and nutrient limitation along the main axis of the bay, wherein high SRF and low salinity in the OH salinity zone in wet years were accompanied by high turbidity, leading to reduced euphotic-layer *chl-a* and NPP. Conversely, high SRF and lower salinities in MH and PH salinity zones were accompanied by increased N-throughput and fertilization of seaward regions of the bay, leading to higher euphotic-layer *chl-a* and NPP in wet years.

## Discussion

Land-margin ecosystems are among the most productive on Earth, contributing disproportionately by area to secondary production and fisheries yields^34^. Biotic richness of these ecosystems hinges on high phytoplankton biomass and primary productivity, driven by nutrient loadings from anthropogenic and natural sources. Intense land-use in bordering watersheds often results in nutrient over-enrichment, producing symptoms of water-quality degradation such as high *chl-a*, reduced water clarity, and low dissolved oxygen (DO) in bottom waters^3^,^4^. Historical evidence of ecosystem responses to human activities in Chesapeake Bay is chronicled in stratigraphic signatures spanning several centuries^35^,^36^. During the 20^th^ century alone, TN and TP loadings to the bay increased 6- and 17-fold, illustrating the rate of cultural eutrophication^10^.

Water-quality trends in the bay have been described before: (1) Murphy *et al.*^15^ reported improving DO based on hypoxic volume, density stratification, and TN loading from 1985–2009; (2) Zhou *et al.*^16^ showed no significant DO trend, with climatic conditions explaining inter-annual variability of hypoxic volume; (3) Prasad *et al.*^37^ estimated trends from gridded time-series data for *chl-a*, nutrients, and freshwater flow using monitoring data from 1985–2008; (4) Williams *et al.*^38^ computed biotic metrics for the main stem bay and its tributaries from 1986–2008. These studies were restricted to monitoring data collected since the mid-1980s, however, and did not provide a historical perspective for the period prior to the most serious ecosystem impairments.

Here, we accessed long-term data for Chesapeake Bay covering earlier decades when cultural eutrophication accelerated, leading to multiple ecosystem impairments by the 1980s^3^. This approach allowed us to extend the analysis to a period when TN and NO_2_ + NO_3_ loadings were less than half the maximum values in the time series (Fig. 3). Our reasoning for focusing on TN and NO_2_ + NO_3_ loadings was that N limits phytoplankton growth and production on an annual scale in the bay^39^. We found that flow-adjusted TN loading reached a maximum of ~69 (×10^6^) kg yr^−1^ in the mid-1980s and declined to ~53 (×10^6^) kg yr^−1^ by 2012 (Fig. 3a). Flow-adjusted NO_2_ + NO_3_ loadings reached a maximum of 45 in the late-1980s to early-1990s and declined to 39 (×10^6^) kg yr^−1^ by 2012 (Fig. 3b). Despite progress toward nutrient reductions in recent decades, annual TN and NO_2_ + NO_3_ loadings have yet to reach thresholds of 40 and 28 (×10^6^) kg yr^−1^, respectively, deemed necessary to achieve bottom-water DO >1 mg L^−1 ^2,14. Current loadings are also higher than unmet goals transcribed in the Chesapeake Bay Agreement for the year 2000^8^. Clearly, more aggressive action is required to counteract the steep increases of TN and NO_2_ + NO_3_ loadings after World War II.

Complicating analyses of long-term trends, climatic conditions produce irregular dry and wet years, leading to high variability of annual SRF and nutrient loadings in the past several decades (Figs. 2 and 3). These conditions generate ecosystem-scale responses of phytoplankton dynamics observed as contrasting *chl-a*, floral composition, and NPP during dry and wet years. Here, we argue that distinguishing long-term trends of phytoplankton dynamics from variability traceable to climatic conditions is essential to quantify secular changes. Specific results for nutrient loadings (Fig. 3), *chl-a* (Figs. 4 and 5), floral composition (Figs. 6 and 7), and NPP (Figs. 8 and 9) support this argument.

Climatic conditions strongly affect nutrient loadings, with important ramifications for phytoplankton dynamics. N inputs are higher during wet years, extending the areal extent of N-sufficiency seaward to MH and PH salinity zones. Conversely, N inputs are lower during dry years and N-limitation occurs further landward in the MH salinity zone. Long-term data on surface *chl-a* from shipboard and aircraft measurements spanning several decades show the effects of climatic conditions on spatio-temporal distributions of phytoplankton biomass (Figs. 4 and 5). Climatic conditions also affect inputs of bio-optically active constituents, with higher inputs of dissolved and suspended materials in wet years than dry years, influencing light penetration as K_D_ (PAR)^2^. The spatial extent of light- and N-limitation is expressed in distributions of surface *chl-a*, floral composition, and NPP that reflect strong forcing by climatic conditions^13^,^17^,^20^.

Combined data from satellite ocean color (SeaWiFS), shipboard surveys, and aircraft remote sensing have been used to document effects of climatic conditions on inter-annual variability of *chl-a* in waters of the bay and adjacent W. Atlantic shelf^18^,^30^,^40^. Wet years with high annual SRF deliver more nutrients to the bay and support higher *chl-a*, while dry years with low annual SRF deliver less nutrients and support lower *chl-a* (Figs. 2, 3, 4). Wet years with higher *chl-a* also show changes of floral composition as the area of N-sufficiency is expanded seaward, expressed as a higher proportion of diatoms (Fig. 6), and accompanied by a rightward shift of the cell-size distribution (Fig. 7)^20^. Accordingly, management-driven derivations of numerical *chl-a* criteria for Chesapeake Bay encompass regulatory goals and thresholds for low-flow, mid-flow, and high-flow conditions, separating short-term effects on *chl-a* traceable to climatic conditions from long-term trends signaling responses to nutrient reductions^12^.

Wet years show a higher frequency of sea-level pressure patterns corresponding to warm, wet conditions, coinciding with high annual SRF, increased nutrient delivery, and higher spring-bloom *chl-a* in MH and PH salinity zones as nutrient-limitation is alleviated. Conversely, dry years show a higher frequency of sea-level pressure patterns corresponding to cool, dry conditions, coinciding with low annual SRF, decreased nutrient delivery, and lower spring-bloom *chl-a* in these same salinity zones (Fig. 4b,c)^18^. This fertilizing effect of high SRF along the landward-seaward axis of the bay expresses a mix of hydrological effects on nutrient loadings and bio-optical properties that strongly influences phytoplankton dynamics. A notable example of this interplay of light and nutrients is evident as higher *chl-a* observed in MH and PH salinity zones following the phosphate detergent ban in the 1970s, resulting from increased N-throughput and a seaward extension of N-sufficiency^2^. Such effects on the *chl-a* distribution reflect slow, non-linear responses to gradual decreases of N loadings since the early 1980s: increased P-limitation now supports lower *chl-a* in the OH salinity zone, allowing more N to reach MH and PH salinity zones where ambient light conditions are conducive to phytoplankton growth.

Non-linear trajectories for recovery of Chesapeake Bay to previous conditions are likely to resemble complex responses of phytoplankton dynamics to nutrient reductions in a number of estuarine and coastal ecosystems^41^,^42^. Based on results for other ecosystems where management actions have successfully reduced nutrients, we cannot anticipate significant decreases of *chl-a*, diatoms, and NPP with the modest decreases of TN and NO_2_ + NO_3_ loadings to the bay that have been achieved thus far. Nor do we anticipate decreases of *chl-a*, diatoms, and NPP will follow linear trajectories and return directly to past conditions. Changes in the efficiency of biomass production expressed as increased *chl-a* / TN in OH and MH salinity zones since 1995 is consistent with observations for recovering ecosystems^2^. This is likely because other biotic resources in the bay have changed over the past 60–70 years, among them precipitous declines of sea grasses, eastern oysters, and Atlantic menhaden^3^. The loss of these components of an intricate food web, combined with recent water-quality improvements, lend stability to mean annual surface *chl-a*, manifested as relatively constant flow-adjusted values in the PH salinity zone and a continuing, gradual increase in the MH salinity zone (Fig. 4b,c). This stability, once climatic conditions are taken into account, reflects multiple influences on phytoplankton dynamics in the bay, rather than resilience.

Comparable effects of climatic conditions on phytoplankton dynamics to those reported here occur in estuarine and coastal ecosystems around the world^22^,^23^,^24^,^25^,^26^,^27^,^28^,^29^,^30^. For Chesapeake Bay, water-quality properties were related to indices of the North Atlantic Oscillation (NAO) and El Niño – Southern Oscillation (ENSO), revealing significant relationships of *chl-a* and freshwater flow to NAO and ENSO that explained only 0.25 to 23% of the variances^37^. We found a synoptic climatology^31^ proved more effective to explain inter-annual variability of phytoplankton, zooplankton, and fish than these basin-scale indices^18^,^32^,^33^,^43^,^44^, although neither approach produced a quantitative solution separating long-term trends from variability imposed by climatic conditions. The approach presented here accounts for variable climatic conditions by generating flow-adjusted outputs using GAM, thereby accomplishing that separation.

Variable climatic conditions significantly affect euphotic-layer *chl-a* and NPP, reflecting effects of annual SRF on distributions of light and nutrients in the bay (Figs. 8 and 9; Table 3). Wet years with high annual SRF experience high N-loading and an increased spatial extent of high K_D_ (PAR), while dry years with low annual SRF experience opposite effects on N loading and K_D_ (PAR)^2^. These patterns were discussed in an earlier study based on a subset of data (1982–1998), defining annual cycles of euphotic-layer *chl-a* and NPP that were characterized by a spring *chl-a* maximum and a subsequent summer NPP maximum^13^. NPP data from that study supported estimates of AIP ranging from 282 to 538 g C m^−2^ yr^−1^ 13, values that were consistent with outputs of a depth-integrated model (DIM) applied to remotely sensed observations that estimated AIP ranging from 400 to 500 g C m^−2^ yr^−1^ 33. Coincident forcing of euphotic-layer *chl-a* and AIP by climatic conditions was evident in the significant, linear relationship of AIP to annual mean euphotic-layer *chl-a* that explained ~62% of the variance^33^. AIP values of this magnitude place the bay among ‘eutrophic’ ecosystems based on the classification scheme of Nixon^45^.

Sensitivity of *chl-a*, floral composition, and NPP to climatic conditions has consequences for secondary production in Chesapeake Bay^46^. Wet years with higher *chl-a*, diatoms, and NPP correspond to enhanced recruitment of Atlantic menhaden, an important Clupeid fish in the eastern United States. Since the late 1980s, age-0 menhaden have been more abundant in years of high phytoplankton biomass, particularly from April-June when juvenile menhaden acquire the ability to filter feed and consume phytoplankton cells. Although frequently hypothesized, such relationships between *chl-a*, NPP, and recruitment levels for marine fishes have rarely been confirmed^47^,^48^. These findings demonstrate that variable climatic conditions dominating phytoplankton dynamics may have a pervasive effect on the food web, consistent with Ryther’s seminal paper linking photosynthesis to fish production in major ocean provinces^34^.

## Conclusions

We demonstrate here that climatic conditions affect phytoplankton dynamics in Chesapeake Bay in predictable ways, captured in a conceptual model that synthesizes our findings (Fig. 10). Summarizing:

- Flow-adjusted N-loadings provide a measure of the trajectory of eutrophication and changes since management actions were put in play;
- Time series of *chl-a* exhibit features reflecting both management actions (P-ban, OH salinity zone), and ecosystem-scale effects (N-throughput, MH and PH salinity zones) suggesting a non-linear recovery path;
- Climatic conditions affect floral composition, with higher diatom abundance and a shift toward a larger cell-size distribution during wet years;
- Primary productivity as NPP is higher during wet years, with ramifications for secondary production and fish recruitment;
- Together, climatic conditions act on the ecosystem in predictable ways through effects on phytoplankton dynamics.

## Methods

### Freshwater flow, nutrient loading

Our analyses were based on data from 1945–2015 from multiple sources. Water-quality properties included annual N loadings (TN, NO_2_ + NO_3_) from the Susquehanna River at the Conowingo Dam gaging station. These data were obtained from two sources: Hagy *et al.*^14^ for 1945–2001 and the US Geological Survey (USGS) Non-tidal Monitoring Program (http://cbrim.er.usgs.gov/) for 1981–2012^49^. Annual SRF from 1945–2015 was computed from daily discharge (ft^3^ d^−1^) converted to metric units (m^3^ d^−1^) and summed over time (10^9^ m^3^ yr^−1^). Climatic conditions were categorized as dry, long-term average (LTA), or wet using annual SRF and a synoptic climatology for the mid-Atlantic region^31^,^32^. Flow-based categorizations were based on long-term mean annual SRF from 1945–2015 of 34.1 (×10^9^) m^3^ yr^−1^, with 25^th^ and 75^th^ percentiles used to delineate dry and wet years, respectively (Fig. 2). We developed a synoptic climatology in previous studies using frequencies of 10 sea-level pressure patterns based on data from the National Climate Data Center (NCDC)^18^,^32^,^33^,^43^,^44^. Designations of individual years as dry, LTA, or wet using annual SRF and the synoptic climatology agreed and water-quality data were aggregated using these categories.

### Water-quality properties

**Figure 1:**
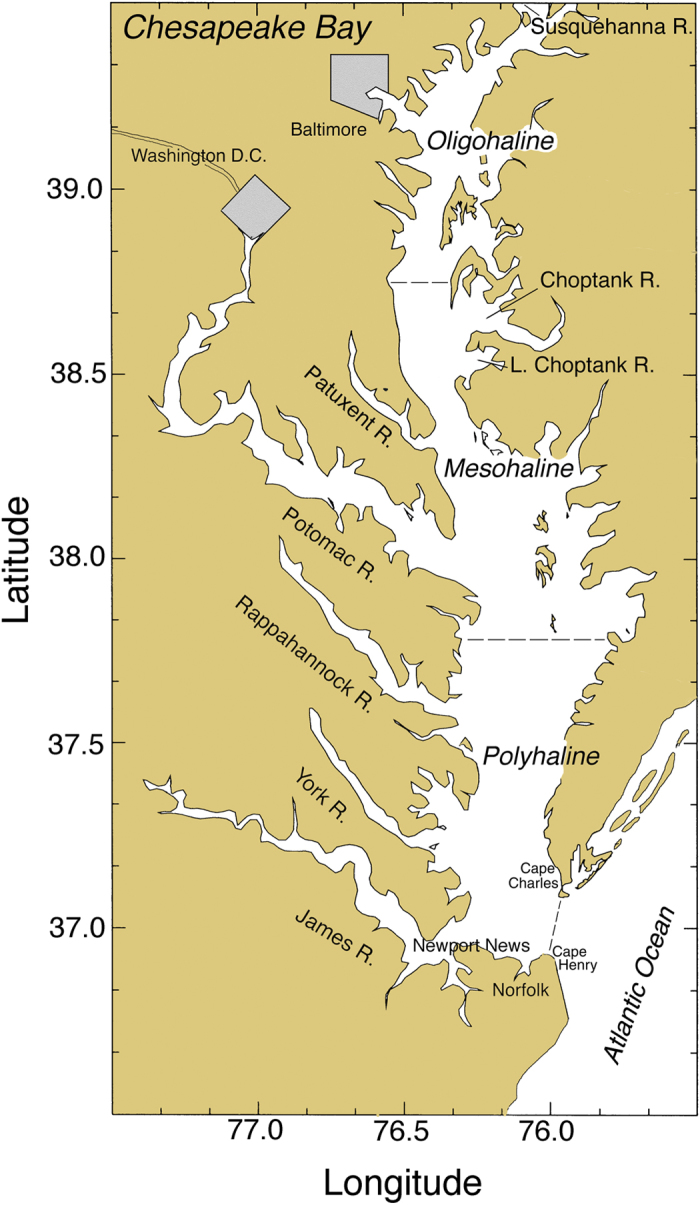
Chesapeake Bay showing major rivers, cities, salinity zones, and sampling stations for pigments and cell counts. Study-site map was generated using the software package Surfer v. 8 (Golden Software; URL http://www.golden software.com) customized with Photoshop v. CS6 (Adobe; URL http://www.adobe. com).

Data on salinity (S), temperature (T), and *chl-a* for the bay’s main stem were obtained from the historical archive of US EPA Chesapeake Bay Program (CBP) contributed by the Chesapeake Bay Institute (1950–83), cruises aboard the R/V *Cape Hatteras, Ridgely Warfield*, and *Cape Henlopen* (1982–88), and the CBP Monitoring Program (1985-2015). Sampling stations were distributed in OH, MH, and PH salinity zones (Fig. 1) using published latitudinal boundaries^11^. Comparable methods were used to determine *chl-a* for data included in our analyses. Most measurements used acetone extracts (80–90%) of particulate material collected by vacuum filtration on glass-fiber filters (GF/F or equivalent) with small (0.3–0.8 μm) nominal pore sizes. These extracts were analyzed by spectrophotometry on a Beckman DK-2 or equivalent, and *chl-a* was quantified by trichromatic equations^50^. Fluorometric measurements of *chl-a* were made on a Turner model 110, 111, or Turner Designs model 10 calibrated by spectrophotometry. Harding & Perry^11^ provided additional details on *chl-a* analyses.

### Remotely sensed *chl-a*

Ocean color measurements from aircraft as part of the Chesapeake Bay Remote Sensing Program (http://www.cbrsp.org) were used to obtain remotely sensed *chl-a* data presented in Fig. 5(b). The Ocean Data Acquisition System (ODAS) (NASA) and SeaWiFS Aircraft Simulator (SAS II, III) (Satlantic, Inc.) were deployed on light aircraft ~20–30 times per year on a set of tracks covering the main stem bay, using methods detailed by Miller & Harding^18^ and references therein. Data were gridded and interpolated to 1-km^2^ for visualization and aggregated by climatic conditions.

### Floral composition

Samples to determine concentrations of algal photopigments (n = 540) were collected on spring, summer and fall cruises from 1995–2004. Surface water was vacuum-filtered (<150 mm Hg) onto glass-fiber filters (GF/F or equivalent) to collect phytoplankton. Filters were flash frozen on dry ice, held at −20 °C until return to the laboratory, and stored at −80 °C prior to analysis using high-performance liquid chromatography (HPLC). Van Heukelem *et al.*^51^,^52^ provided additional details for extraction and chromatographic methods. Reconstructions of floral composition from pigment concentrations generated the proportions of *chl-a* in seven major taxonomic groups using CHEMTAX software^53^,^54^. The method relies on concentration matrices of diagnostic carotenoids and chlorophylls to compute abundances. Outputs consisted of proportions of diatoms, cryptophytes, dinoflagellates, cyanobacteria, prasinophytes, two groups of haptophytes (types 7 and 8), and chlorophytes for each group (*f_chl-a*^*taxa*^). Data were aggregated by salinity zone, season, and climatic conditions. Harding *et al.*^20^ provided additional details of floral composition determined using algal photopigments.

Cell counts were obtained from semi-monthly to monthly cruises of the CBP monitoring program from 1985–2007 for nine stations in the bay. Samples were collected in duplicate 20-L carboys using a submersible pump at several equally spaced depths above the pycnocline. Aliquots (500 ml) were withdrawn from each carboy, fixed in buffered Lugol’s solution, and preserved in formalin on return to the laboratory. Cell dimensions for each species were used to compute cell volume (CV) from species-specific shape codes. Corrections were applied to taxonomic groups with vacuoles (e.g., diatoms) to compute plasma volume (PV). Phytoplankton records were obtained from the CBP data hub (http://www.chesapeakebay.net/data), including identifications to species, National Oceanographic Data Center (NODC) database designations, two-digit codes for major taxonomic groups, cell counts, dimensions, CV, PV, and cell carbon (C)^55^. Data were aggregated by salinity zone, season, and climatic conditions. Harding *et al.*^20^ provided additional details on methods for estimating floral composition from cell counts and dimensions.

### NPP

NPP was measured on cruises from 1982–2004 using 24-h ^14^C-bicarbonate assimilation on 723 individual samples collected at sunrise in Niskin bottles on a rosette sampler. Vertical profiles of salinity, temperature, DO, and *chl-a* fluorescence were conducted at each station using Yellow Springs salinity and oxygen meters (1982–1983), a Sea-Bird 9 CTFO2 (1987–1988), a Neil Brown Mark III CTDFO2 (1989–1996), and a Sea-Bird SBE911 plus (1997–2004). Discrete samples were used to calibrate the fluorometers and oxygen meters and to measure NPP. Contents of Niskin bottles were pooled in a darkened carboy, dispensed to 125–150 ml glass incubation bottles, and each bottle was amended with 2 to 5 μCi of ^14^C-sodium bicarbonate (ICN Pharmaceuticals, Inc., or Amersham Searle, Inc.). Total activity was determined from a time-zero aliquot withdrawn from an incubation bottle and as the activity of a small amount of stock isotope added directly to scintillation cocktail (Aquasol, New England Nuclear, Inc., or equivalent) made basic with NaOH.

Bottles were placed in simulated *in-situ* sunlight incubators on the ship’s upper deck and cooled with flowing surface water. Layers of neutral density screens allowing 58, 34, 21, 11, 4 and 1% transmission were used to attenuate sunlight, and clear bottles provided 100% transmission. Dark uptake was measured in an opaque bottle to correct all measurements. Surface sunlight (E_0_ = downwelling irradiance, photosynthetically available radiation = PAR) was measured with a Li-Cor model 190S quantum meter (or equivalent) mounted near the deck incubators and coupled to a Li-Cor model 550 or 1000 integrator. The diffuse light attenuation coefficient for PAR, K_D_ (PAR), was calculated from vertical profiles of downwelling irradiance, E_D_, using a Li-Cor model 188B quantum meter with a 192S sensor (or equivalent). Coincident readings of Secchi depths were made at all stations. The depth of the euphotic layer, Z_p_, was determined from vertical profiles as the depth to which 1% E_0_ penetrated, or estimated from Secchi depth using published corrections for Chesapeake Bay^56^.

Aliquots of 25–150 ml (depending on phytoplankton biomass) were vacuum-filtered onto glass-fiber filters (Whatman GF/F or equivalent) at the end of incubations. Filters were rinsed several times with filtered water of equivalent salinity and gently acidified with 0.01 N HCl in a fume hood to remove residual inorganic label. Activities were determined on a Packard Instruments Tri-Carb or model 3320 liquid scintillation counter. We measured total CO_2_ by gas stripping, capture, and analysis on a Beckman model 864 infrared analyzer (1982–83), Gran titration (1987–1988), or gas chromatography using a Hach Cable Series 100 AGC (1989–2004). NPP was measured in duplicate bottles, and integrated, daily production (g C m^−2^ d^−1^) was computed by trapezoidal integration of NPP values over depth. Harding *et al.*^13^ provided additional details on methods to measure NPP.

### Statistical asnalyses

Statistical analyses used the “Pumpkin Helmet” version of R. Mann Kendall tests from the R package ‘wq’^57^ were applied to time-series data consisting of mean annual values of SRF, N loadings (TN and NO_2_ + NO_3_), and *chl-a*. Non-linear fits were developed with time-series data using GAM from the ‘mgcv’ package^58^,^59^ and GAMM from the ‘gamm’ package^60^. The ‘mgcv’ package in R is similar to GAM functions in S-Plus designed by Trevor Hastie, but is based on a penalized regression-spline approach with automatic smoothness selection. The gamm package allowed us to test for lag effects by including an auto-regressive (AR) term. ANOVA were run in S-Plus v. 6.2.

Model fits, residuals, flow-adjusted values as predictions at half-yearly increments, model R^2^, generalized cross validation (GCV) score, % deviance explained, and p-values for F-statistics were obtained for each model. Models were applied to log_10_ annual SRF held constant at its long-term mean for the period covered by the data to obtain flow-adjusted values. Degrees of smoothing (knots = k) were selected by the package to minimize the GCV score. Time-series data were presented as mean annual values (±SE) accompanied by model fits and flow-adjusted values. Trends for specific periods were computed as percent changes based on flow-adjusted outputs. Harding *et al.*^2^ provided additional details on trend analyses.

**Figure 2:**
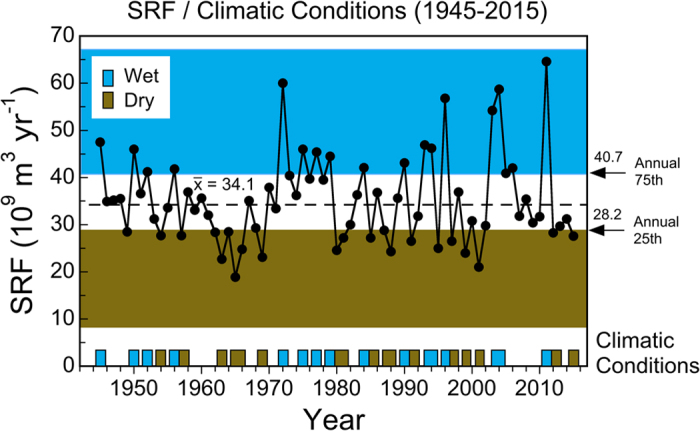
Annual freshwater flow from the Susquehanna River for 1945–2015, indicating dry and wet years based on 25^th^ and 75^th^ percentiles.

**Figure 3:**
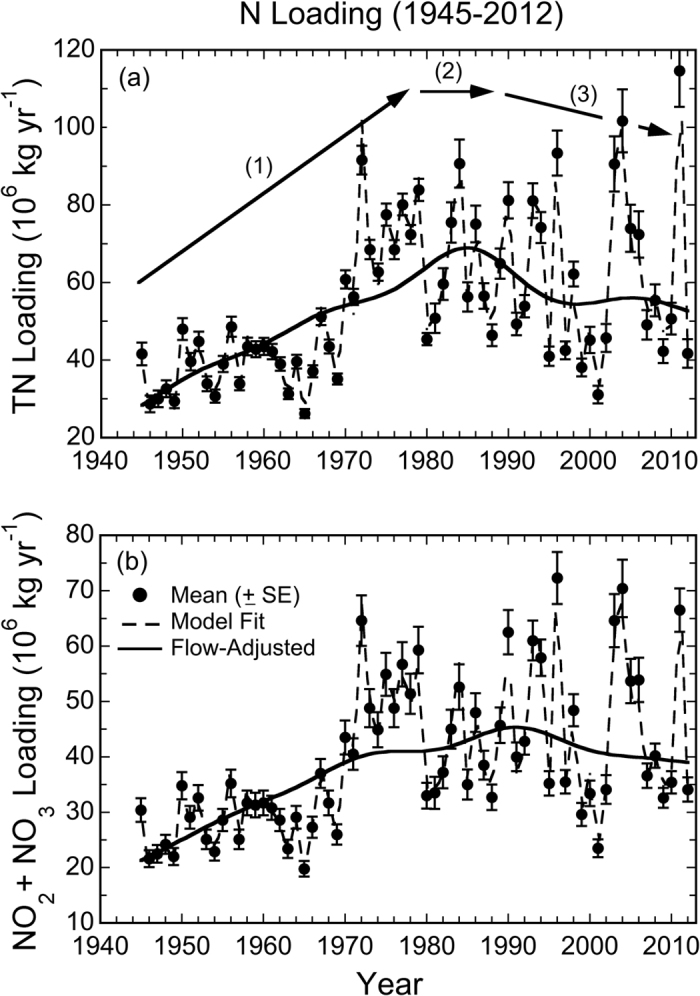
(**a,b**) Time-series (1945–2012) of mean (±SE) annual N loadings (TN and NO_2_ + NO_3_) at the Conowingo Dam. Data from Hagy *et al.*^14^ (open circles, 1945–1980), and USGS (closed circles, 1981–2012)^52^. Dashed lines depict GAM fits; solid lines depict flow-adjusted GAM outputs. Annotations on panel (**a**) refer to: (1) period of increasing TN loading from 1945 to 1980; (2) leveling off ca. 1980; (3) decreasing TN loading from 1981–2012. Adapted from Harding *et al.*^2^.

**Figure 4:**
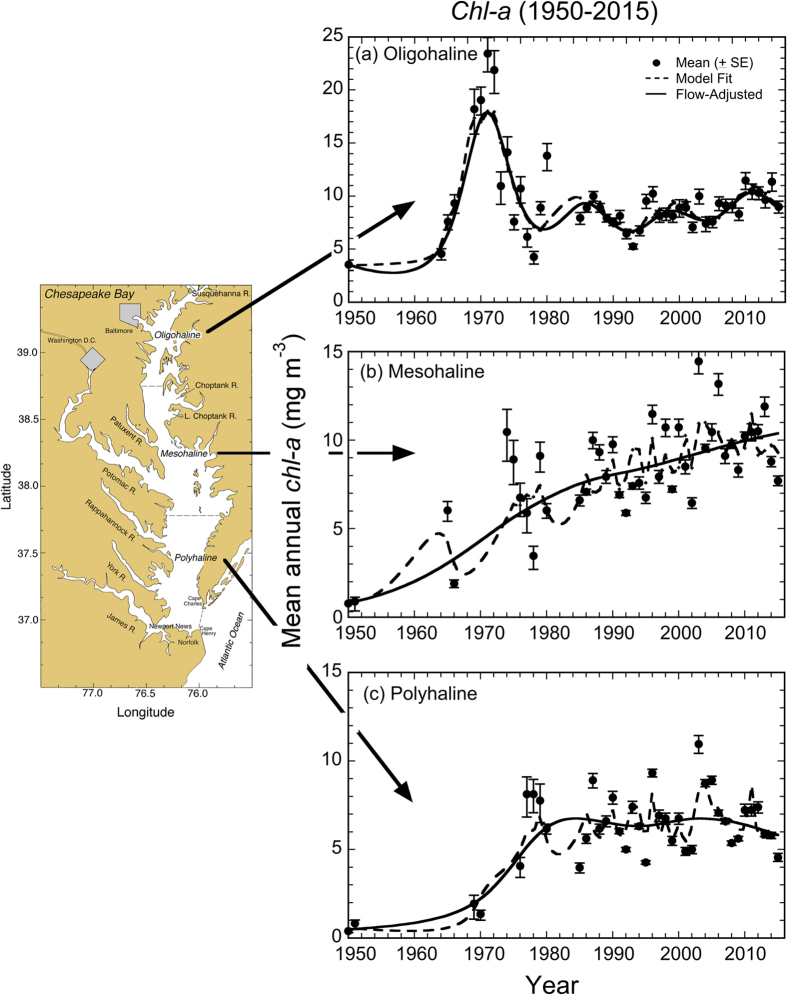
(**a–c**) Time-series (1950–2015) of mean (±SE) annual surface *chl-a* concentrations for OH, MH, and PH salinity zones. Dashed lines depict GAM fits; solid lines depict flow-adjusted GAM outputs. The study-site map was generated with software package Surfer v. 8 (Golden Software; URL http://www.golden software.com) customized with Photoshop v. CS6 (Adobe; URL http://www.adobe. com). Updated from Harding *et al*.^2^ with additional data.

**Figure 5:**
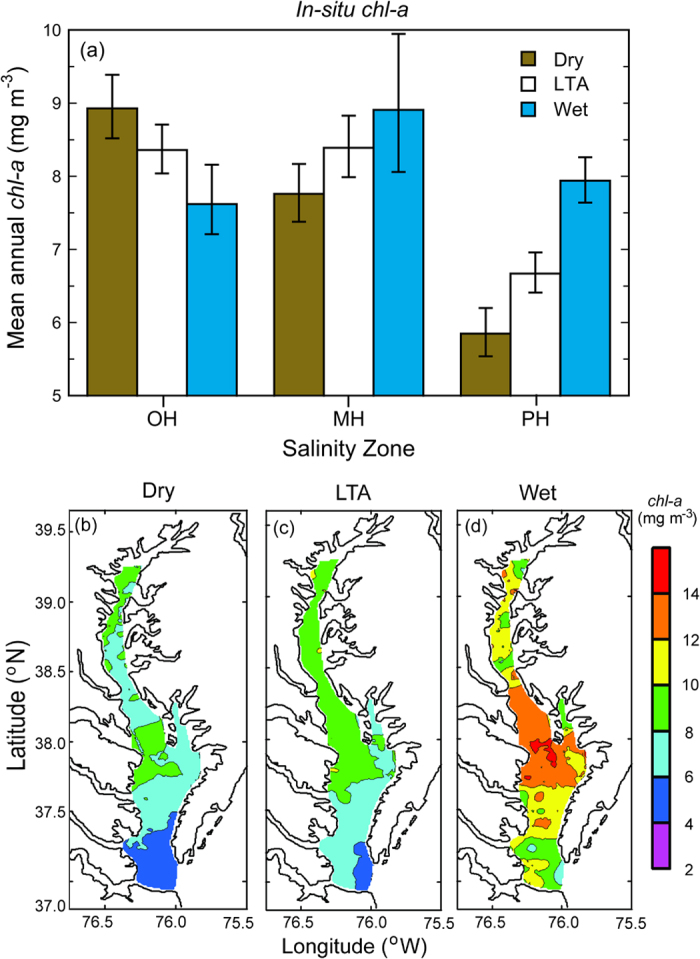
(**a**) Mean annual *chl-a* (±SE) aggregated by climatic conditions using *in-situ* observations for OH, MH, and PH salinity zones from 1950–2015; (**b–d**) remotely sensed *chl-a* during the spring bloom from aircraft remote sensing 1989–2005. *In-situ chl-a* was measured on shipboard surveys, and remotely sensed *chl-a* on low-altitude flights using ocean-color sensors as part of the Chesapeake Bay Remote Sensing Program (CBRSP) (see Methods). Plots of remotely sensed *chl-a* aggregated by climatic conditions were produced using the software package Surfer v. 8 (Golden Software; URL http://www.golden software.com) customized with Photoshop v. CS6 (Adobe; URL http://www.adobe. com).

**Figure 6:**
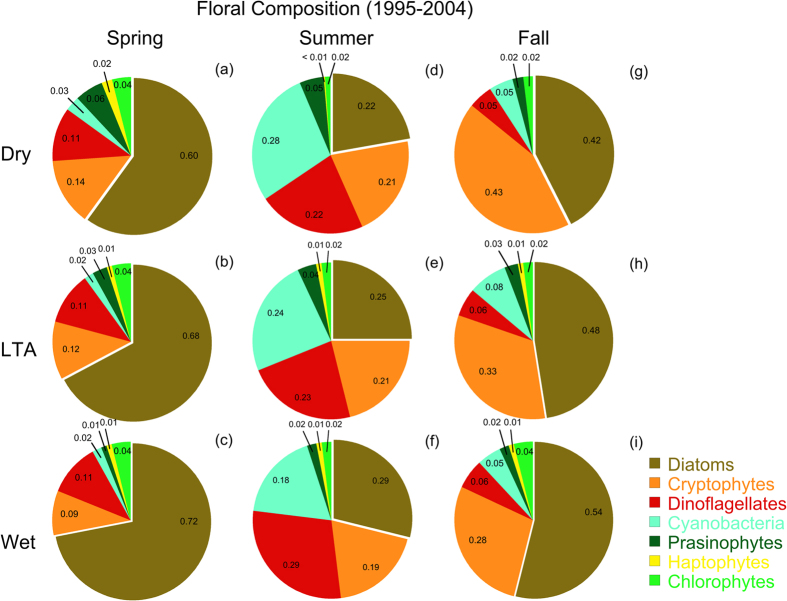
(**a,b**) Pie plots of floral composition as proportions of *chl-a* (*f_chl-a*^*taxa*^) based on HPLC analyses of algal photopigments aggregated by dry, long-term average (LTA) and wet climatic conditions. Results are presented for spring, summer, and fall using combined data for all three salinity zones. See Harding *et al*.^20^ for further details.

**Figure 7:**
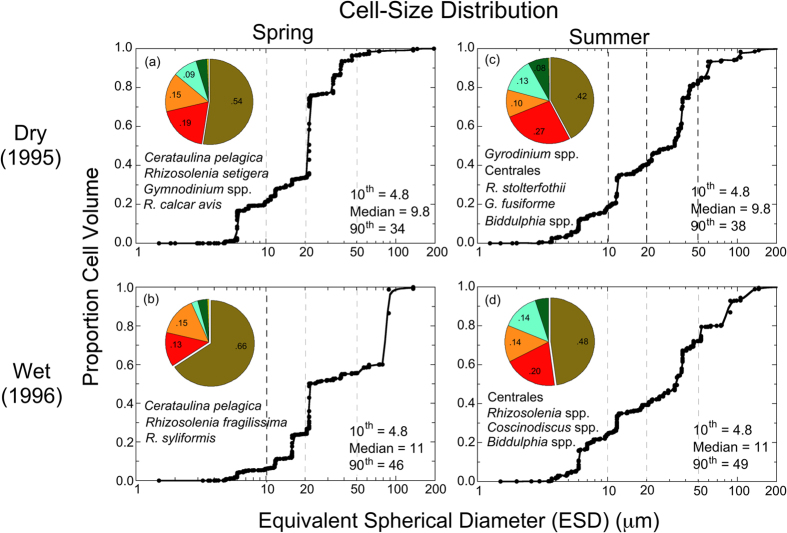
(**a–d**) Proportion of total cell volume vs. equivalent spherical diameter (ESD) in spring and summer for consecutive dry (1995) and wet (1996) years. Horizontal arrows indicate shifts of cell-size distributions to higher proportions of cell volume at smaller ESD during dry years, and higher proportions of cell volume at larger ESD during wet years. Pie-diagram inserts show proportions of major taxonomic groups and prominent species in the respective samples. Adapted from Harding *et al*.^20^.

**Figure 8:**
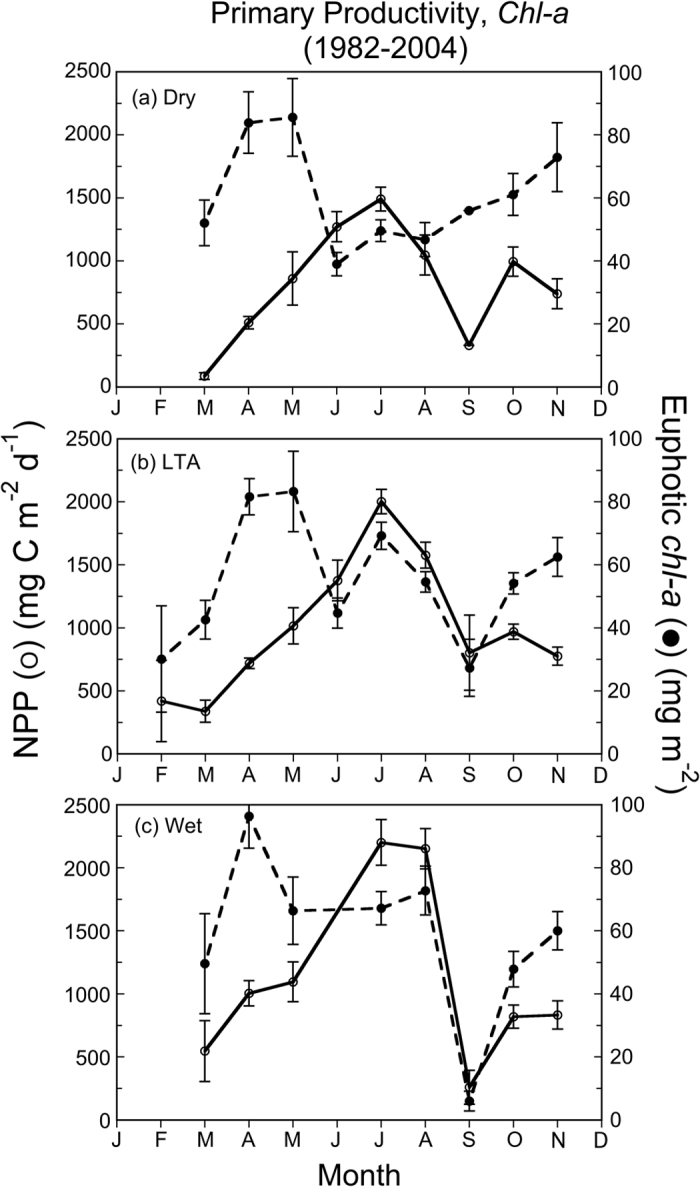
(**a–c**) Monthly means (±SE) of net primary productivity (NPP) (left y-axis) and euphotic-layer *chl-a* (right y-axis) aggregated by dry, LTA, and wet climatic conditions for 1982–2004.

**Figure 9:**
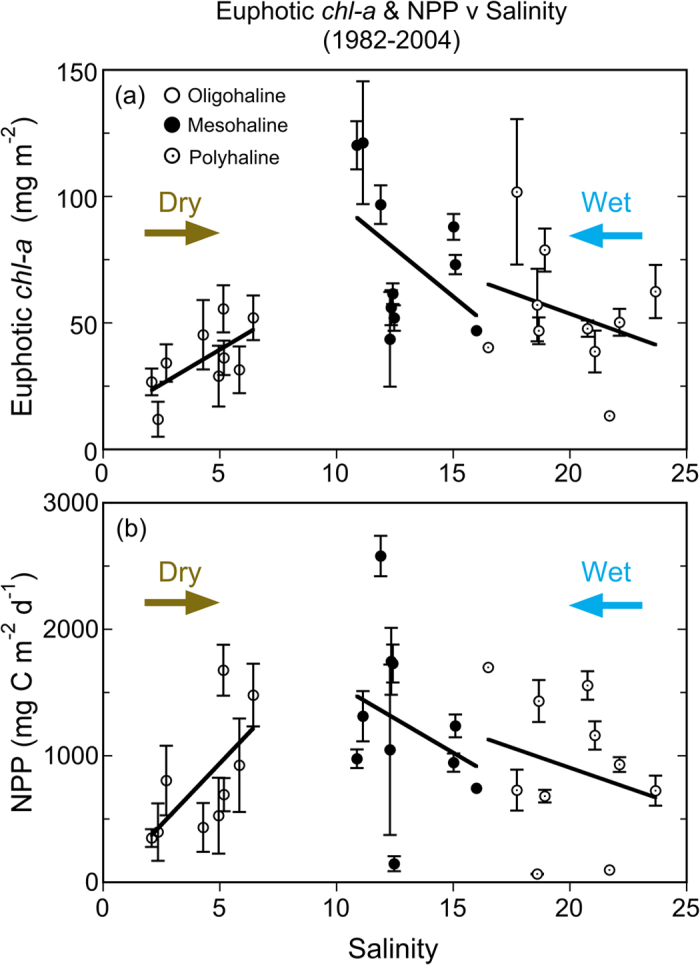
(**a**) Euphotic-layer *chl-a* and (**b**) NPP vs. salinity for data aggregated by salinity zone in the main stem bay for 1982–2004. Horizontal arrows and labels illustrate the effect of dry and wet conditions on the distributions of euphotic-layer *chl-a* and NPP.

**Figure 10:**
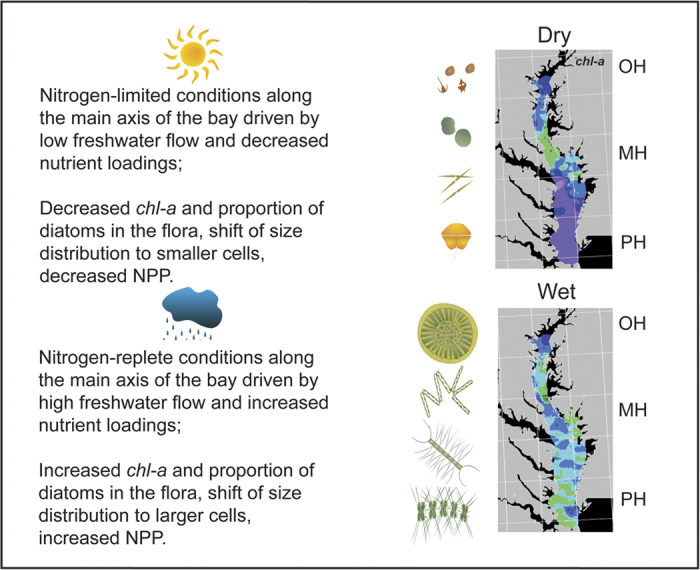
Conceptual model of climatic effects on phytoplankton dynamics in Chesapeake Bay. Text inserts summarize effects of dry and wet climatic conditions. Paired images from aircraft remote sensing of *chl-a* in mid-April illustrate the effect of N-depletion on the seaward extent of high *chl-a* during a dry year (2009) (upper panel) compared to a wet year (2003) (lower panel). Algal cartoons represent the lower *chl-a* biomass, lower proportion of diatoms, and lower NPP during dry years and higher *chl-a* biomass, higher proportion of diatoms, and increased NPP during wet years. Symbols courtesy of the Integration and Application Network, University of Maryland Center for Environmental Science (http://ian.umces.edu/symbols/). Remotely sensed data were plotted using custom programs developed by the authors (http://www.cbrsp.org). The conceptual model was designed using the software Illustrator v. CS6 (Adobe; URL http://www.adobe. com).

Chesapeake Bay maps (Figs. 1 and 5) were produced using Surfer (Golden Software), and customized with Adobe Photoshop. Processing of original remote sensing images of *chl-a* used custom software written by the authors in Fortran 77 (http://www.cbrsp.org), and modified for presentation using Adobe Illustrator and Adobe Photoshop. Other graphics (Figs. 2, 3, 4, 5, 6, 7, 8, 9) were developed with Kaleidagraph (Synergy Software). Symbols for the conceptual model were provided by the symbol library of the Integration and Application Network (IAN) of the University of Maryland Center for Environmental Science (see Fig. 10 legend for attribution).

## Additional Information

**How to cite this article**: L. W. Harding Jr. *et al.* Variable climatic conditions dominate recent phytoplankton dynamics in Chesapeake Bay. *Sci. Rep.* **6**, 23773; doi: 10.1038/srep23773 (2016).

## Figures and Tables

**Table 1:** Statistics for generalized additive models (GAM) of nutrient loadings and *chl-a* for Chesapeake Bay.

Property	Time frame	R^2^ (adj.)	% Deviance explained	GCV^1^	p-values (year, log_10_ SRF)	Significance^2^
Nutrient Loadings						
TN	1945–2012	0.985	98.8	<0.001	<0.001, <0.001	***, ***
NO_2_ + NO_3_	1945–2012	0.982	98.6	<0.001	<0.001, <0.001	***, ***
*Chl-a*						
OH	1950–2015	0.661	73.9	0.0115	<0.001, 0.334	***, NS
MH	1950–2015	0.811	84.3	0.0159	<0.001, 0.131	***, NS
PH	1950–2015	0.863	88.6	0.0110	<0.001, 0.003	***, ***

Property = log_10_ TN, NO_2_ + NO_3_, or log_10_ *chl-a*; predictor variables = year and log_10_ annual SRF (10^9^ m^3^ yr^−1^).

^1^Generalized cross-validation score.

^2^*p < 0.05; **p < 0.01; ***p < 0.001; non-significant – NS.

**Table 2:** ANOVA results for the four most abundant taxonomic groups of phytoplankton in Chesapeake Bay based on algal photopigments.

Variables	F	p-value	Significance^1^
*f_chl*^*diatom*^			
season	196	<0.0001	***
salinity zone	0.011	0.916	NS
year	6.81	0.009	**
climate	21.5	<0.0001	***
*f_chl*^*crypto*^			
season	80.8	<0.0001	***
salinity zone	6.33	0.012	*
year	0.909	0.341	NS
climate	7.64	<0.001	***
*f_chl*^*dino*^			
season	54.4	<0.0001	***
salinity zone	44.7	<0.0001	***
year	3.64	0.057	NS
climate	0.168	0.845	NS
*f_chl*^*cyano*^			
season	204	<0.0001	***
salinity zone	50.6	<0.0001	***
year	2.55	0.111	NS
climate	15.3	<0.0001	***

^1^Significance: *p < 0.05; **p < 0.01; ***p < 0.001; non-significant – NS.

**Table 3:** Mean (±SE) salinity, *chl-a*, euphotic-layer *chl-a*, and NPP from 1982 to 2004 using data aggregated by climatic conditions.

Salinity zone/Season	Salinity	*chl-a*	Euphotic *chl-a*	NPP
Dry				
OH				
Spring	3.75 ± 0.64	9.91 ± 1.41	40.7 ± 6.65	684 ± 247
Summer	7.84 ± 0.92	11.4 ± 1.67	53.5 ± 8.70	1383 ± 154
Autumn	6.31 ± 1.23	8.71 ± 3.43	36.3 ± 13.8	550 ± 256
MH				
Spring	12.3 ± 0.31	18.3 ± 2.61	114 ± 12.4	576 ± 79.3
Summer	14.3 ± 0.19	8.17 ± 0.60	53.0 ± 2.98	1585 ± 145
Autumn	17.5 ± 0.27	10.2 ± 0.88	80.2 ± 4.10	1118 ± 121
PH				
Spring	19.1 ± 0.57	11.0 ± 1.67	79.1 ± 12.1	528 ± 64.5
Summer	21.6 ± 0.52	6.43 ± 0.47	38.9 ± 2.31	1181 ± 83.3
Autumn	24.3 ± 0.60	10.1 ± 1.79	66.8 ± 10.1	875 ± 111
LTA				
OH				
Spring	3.20 ± 0.52	8.86 ± 0.93	32.0 ± 4.11	555 ± 118
Summer	5.41 ± 0.55	12.2 ± 1.30	51.9 ± 6.49	1563 ± 149
Autumn	5.10 ± 0.67	7.90 ± 1.14	33.7 ± 5.51	659 ± 108
MH				
Spring	11.2 ± 0.28	18.8 ± 1.97	114 ± 10.0	1035 ± 85.0
Summer	12.1 ± 0.23	15.4 ± 1.52	82.3 ± 5.15	2253 ± 117
Autumn	15.0 ± 0.42	9.78 ± 0.55	74.4 ± 3.28	1163 ± 73.0
PH				
Spring	18.5 ± 0.57	11.6 ± 1.27	79.0 ± 7.99	656 ± 48.2
Summer	20.3 ± 0.43	7.41 ± 0.45	46.8 ± 2.61	1493 ± 86.7
Autumn	22.6 ± 0.54	8.13 ± 0.84	53.3 ± 4.90	864 ± 56.8
Wet				
OH				
Spring	1.52 ± 0.42	9.62 ± 1.79	27.9 ± 7.13	389 ± 87.1
Summer	3.11 ± 0.59	13.9 ± 1.68	48.0 ± 7.19	1622 ± 234
Autumn	2.18 ± 0.45	7.51 ± 1.08	22.8 ± 4.54	467 ± 102
MH				
Spring	9.49 ± 0.37	16.1 ± 1.79	98.8 ± 9.57	1437 ± 117
Summer	10.1 ± 0.29	13.7 ± 1.14	81.0 ± 5.43	2555 ± 198
Autumn	11.2 ± 0.59	10.1 ± 1.20	73.3 ± 7.76	1066 ± 124
PH				
Spring	18.5 ± 1.39	21.0 ± 3.32	127 ± 13.7	967 ± 92.2
Summer	17.6 ± 0.76	10.6 ± 1.46	71.3 ± 8.27	2091 ± 220
Autumn	19.4 ± 1.06	8.21 ± 0.79	49.0 ± 5.01	839 ± 92.9
